# Digitized ASIC (Adaptation, Standardization, Integration, and Compliance) Framework: An Innovation for Optimizing Technologies and Innovations for Medical and Higher Education

**DOI:** 10.7759/cureus.77191

**Published:** 2025-01-09

**Authors:** Joshua Owolabi

**Affiliations:** 1 Department of Biomedical Sciences, Philadelphia College of Osteopathic Medicine, Moultrie, USA

**Keywords:** adaptation, artificial intelligence (ai), asic, compliance, framework, higher education, innovations, integration, medical education, standardization

## Abstract

This article serves several specific purposes: Presenting transferable information on transforming innovative ideas into educational products with practical applications; showcasing a digitized version of a leading innovation aimed at optimizing technologies and innovations in medical education, health professions education, and higher education; advancing an evidence-based approach to integrating innovations into educational ecosystems and promoting education through innovations and technologies. A 10-step approach to developing the ASIC framework (ASIC stands for Adaptation, Standardization, Integration, and Compliance) is presented, with explanations and illustrations of the processes and activities involved. Empirical evidence and sound principles are provided in support of activities to assure the validity and reliability of methods or procedures. The product of this innovative process is presented and described for the benefit of educators, academic leaders, and industry stakeholders on evidence-based practical approaches to deploying technologies for educational purposes to benefit learners or teachers, and the general society. The ASIC framework's four tenets, which include Adaptation, Standardization, Integration, and Compliance, are clearly defined. Evidence is presented to support ASIC tenets’ roles in deploying educational technologies and innovations, as well as in transformation agendas involving leading changes with innovation. Possible applications of this successful approach to educational change agenda and roles are also presented. Adequate reference is made to a need to premise interventions on relevant theories and principles including the adult learning theory, cognitive load theory, Bloom’s taxonomy, and connectivism. The IDEO model for leading change with innovation is also highlighted. This article could help educators, innovators, and other stakeholders by providing evidence on methodical approaches to developing and deploying useful innovations.

## Introduction

Educational technologies (EdTech) and innovations have become increasingly integral to other education in general, and especially for medical education and health professional education. This is largely a reflection of general advancements in technologies, innovations, and ways of life that have largely been based on technology. It is also a reflection of the transitions from the industrial age to the information age and the emphasis on the use of cutting- or bleeding-edge approaches to driving changes and creating solutions which in turn is largely technology-dependent. More specifically, there are numerous arguments supported by abundant evidence on the importance of technologies and innovations in support of medical education, medical practice, and health professions. In a world, that is being increasingly tech-driven, the culture of technology has significantly permeated medical, professional, and higher education. In fact, imbuing a tech culture into medical training and practice has arguably become a major aspect of emphasis toward training workers and professionals for the current places of work and is very important to meet the needs of the future demands in workplaces. Graduates and professionals without technological skills would not only have lacked vital skills but would also be alien to the emerging culture of work. People’s acceptance or aversions to technology could be complicated but are not impossible to explain. For example, the technology acceptance model (TAM) posits that users are motivated to use technology by three factors, namely, perceived usefulness, perceived ease of use, and attitude toward use [1-3].

There is evidence that technology is increasingly becoming integral to medical education and health service delivery [4-6]. This is true at the discipline level, such as in anatomy [7] and for medical education in general [8]. The COVID-19 pandemic highlighted the significant value of EdTech, digital innovation, and online learning in supporting and sustaining both medical education and healthcare delivery. This successful adaptation served as a crucial eye-opener, demonstrating the essential role of digital resources in advancing medical and health education while enhancing healthcare delivery systems [9-16]. 

Having established the place of innovation and EdTech in today's educational ecosystem, it is important to further highlight the place of technologies and innovations and their continuous deployment for educational purposes. One thing is clear, technology influences not just the use of technologies for educational activities but also the established methods, traditional practices, and, consequently, the culture of education and practice. In other words, technology used for educational purposes could influence the knowledge, skills, and attitudes of not just the learners and trainees but also the educators and trainers as well. This last statement explains why the use of technology would require critical considerations, references to empirical evidence, and adherence to guiding principles and relevant theories. Poor consideration for standard practices, pedagogical principles, and relevant learning theories has resulted in observable heterogeneities in methods of EdTech use and the impacts they produce on learners. This needs to be addressed. It is in line with these realities that the ASIC framework for guiding the use of EdTech and innovation for medical education and its operational frameworks were developed and published [17-19].

This work, therefore, hypothesizes that heterogeneity in types and uses of innovations and technologies would limit their validity and reliability to achieve educational outcomes or competencies with optimal outcomes except that they are used based on guiding principles that are premised on sound educational theories as well as empirical; the evidence is used to form the basis of judgment, and strategies for use and their pedagogical approaches. Furthermore, this work proposes that to optimize the use of an innovation or EdTech to support higher education or medical education, there are three key areas of consideration which include curriculum, pedagogy, and assessment. 

## Technical report

Method

**Figure 1:**
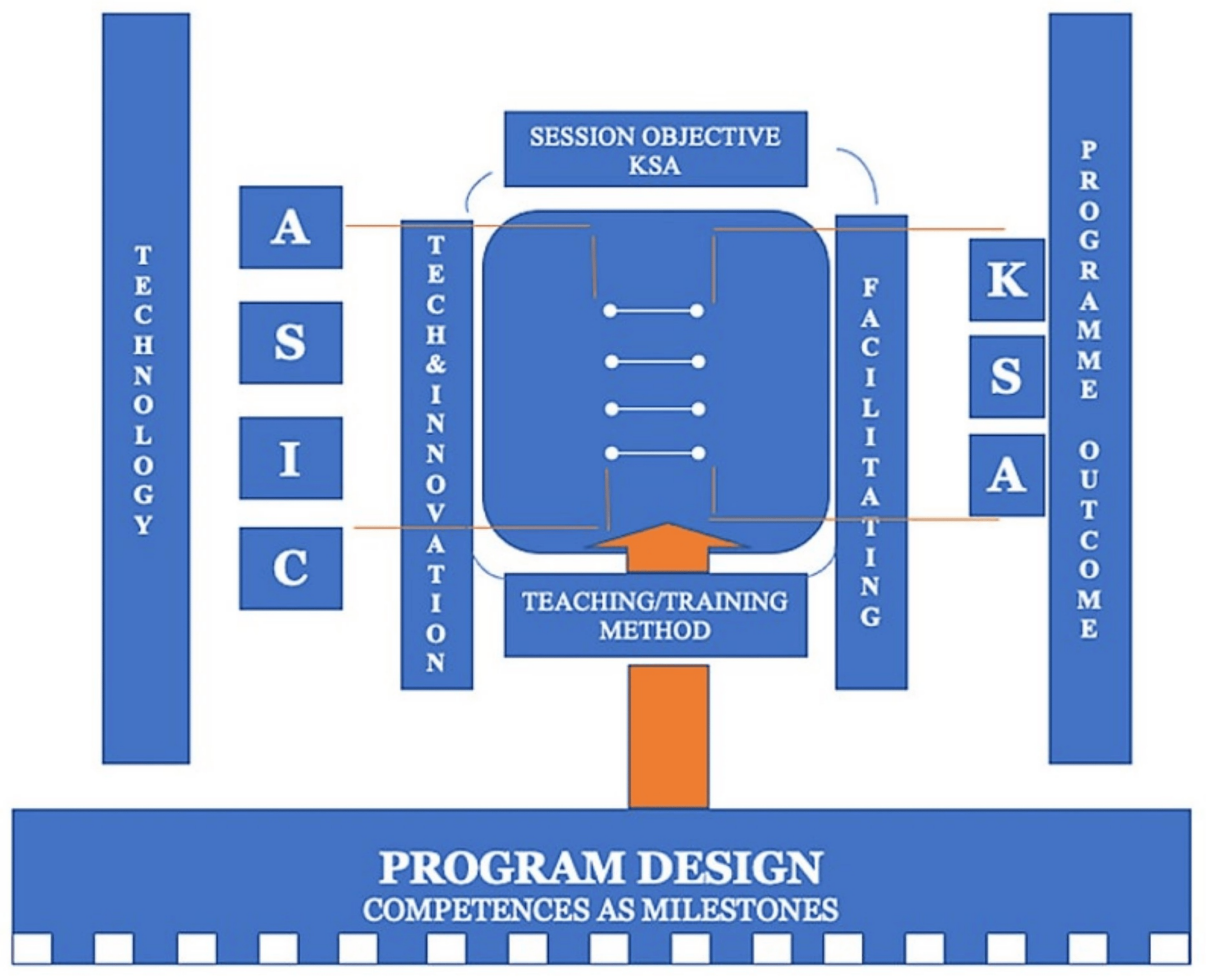
ASIC framework adapted from the original work on ASIC. An illustration showing a relationship between the use of technologies and innovations for educational purposes in a specified context such as the classroom or simulation facility in connection with the outcomes in the domains of knowledge skills and attitude. It also represents the functional and operational relationship between technologies/innovations, teaching or training and program outcomes in relation to competencies as milestones based on program design [17,18]. ASIC: Adaptation, Standardization, Integration, and Compliance; K: Knowledge; S: Skill; A: Attitude.

This section presents a 10-step approach to digitalizing the ASIC framework, starting with defining a clear problem in need of a solution to creating a product with proof of concepts for practical applications and navigating through technical and legal issues. While it is important to state that these steps were not necessarily followed strictly sequentially, it is important to note that the 10 steps have been clearly highlighted in a way that they could form a practical guide for an educator or an innovator seeking information on steps to a methodical approach or producing an educational innovation. They also serve to present evidence that the ASIC framework has been developed with adequate considerations for the creative flow of thought, application of sound medical theories and principles, and project management knowledge and skills (Figure 1). The 10-step approach is highlighted as follows:

Defining a Problem in Need of Intervention

The problem statement for the current work could be stated as follows: Heterogeneities in EdTech and innovation use and impact are resultant of a lack of established standard practices and poor adherence to pedogeological practices and relevant learning theories while deploying educational technology and innovations. The initial idea to have a framework, standard tool, guiding theory, or a set of principles for optimizing the use of innovations and technologies for medical education was identified through experiences, multi-institutional, multinational, and action project activities [20-22]. A critical appraisal of EdTech use in a medical school that was highly innovative and tech-driven yielded a number of considerations that were further crystallized into key tenets. A reflective practice and critical analysis of how the efforts succeeded helped to analyze the purpose of the key tenets. Further critical thinking and analysis helped to design a hypothetical educational ecosystem and connect the tenets with actual elements of the ecosystem, through an iterative process that helped design a sample reference framework model with working principles that could be applied to diverse educational systems. The four tenets include Adaptation, Standardization, Integration, and Compliance. From these, the acronym ASIC was made, and the emergent framework was named the ASIC framework for optimizing EdTech and educational innovations.

Following the successful publication of the original idea as a scholarly article with quality peer review, a tool for operationalizing the framework so that educators and academic leaders can effectively apply the principles of the ASIC framework was developed and published as the ASIC matrix. Continual use of the original matrix provided further insights into a need to further simplify the application of the ASIC principles for medical educators whose interest revolves around certain core aspects of medical or higher education which were defined to include Curriculum (C), pedagogy (P), and assessment (A) in line with the identified pillars of medical education which are relevant to teaching- and training-related practices. Consequently, the ASIC framework operational matrix that addressed innovation and EdTech’s optimization with emphasis on curriculum, pedagogy, and assessment was designed and published as the ASIC framework CPA operational matrix. This ASIC framework CPA operational matrix was successfully digitized for ease of access, use, and appraisal of educational innovations and technologies. It also accrues features that validate the use of the digital tool. 

Establishing a Sound Theoretically Correct and Pedagogically Sound Model

It was important to ensure that the model aligned with relevant learning theories and pedagogical principles for teaching. Here are selected specific instances (see Table 1).

**Table 1:** Steps for establishing a sound theoretically correct and pedagogically sound model. ASIC: Adaptation, Standardization, Integration, and Compliance; EdTech: educational technologies.

	Key considerations	Additional information
a	Adult learning theory [23,24]	ASIC was designed with the premise that the beneficiaries of EdTech use for medical and higher education are adults; it also considers a learner-centered approach.
b	Cognitive load theory [25,26]	ASIC design has a premise that EdTech use should minimize extraneous load and optimize intrinsic load while managing germane load effectively.
c	Miller’s pyramid [27,28]	ASIC framework requires that the learning outcomes should be clearly defined and measurable in line with the EdTech and innovation[s] used.
d	Kolb’s learning cycle [29,30]	The use of an EdTech is aligned with Kolb’s cycle such that it could contribute to the process of achieving a holistic learning experience, e.g. an educator could decide whether an EdTech served the purpose of experimentation or conceptualization.
e	Bloom’s pyramid [31,32]	There is a premise that the use of an EdTech or innovations should be aligned with an identified level of Bloom’s pyramid. For example, an educator should determine whether a learner who uses an interactive 3D anatomy digital atlas needs to identify the already dissected anatomic structures or to explore, self-dissect, identify, and describe.
f	Visual, auditory, reading/textual, and kinesthetic (VARK) modalities [33,34]	Considerations for multiple modality nature of educational resources: There were considerations for the use of resources that provide knowledge (cognitive), or that help to impart skills (psychomotor) or attitude (affective) in the forms of visual, auditory, reading/textual, and kinesthetic (VARK) modalities [33,34].

Generating Practical Evidence From Practice

After putting together ASIC as a framework, evidence was obtained from actual practice in different contexts of subjects, institutions, and students. Publishing the framework first, the first operational matrix and the alternative operational matrix in peer-reviewed articles provided major opportunities for critique from educators and academic leaders. Teaching and communicating the ASIC concepts in professional meetings, institutional training-the-trainers programs, and other fora provided quality feedback for improvement. There was also published evidence of adoption in other climes. 

Designing an Algorithm Flow

**Figure 2:**
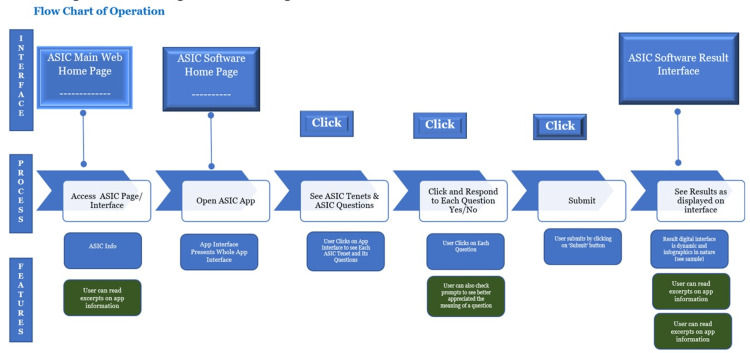
Summary of algorithm flow of actions and functions for the start to submission. The process includes the following: Access Interface -> Open App -> See ASIC Tenets & ASIC Questions -> Click and Respond to Each Question Yes/No --> Submit -> See Results as displayed on the interface. ASIC: Adaptation, Standardization, Integration, and Compliance.

An algorithm flow for the digital operation of ASIC was developed (Figure 2). The algorithm was communicated to a team of assembled designers and programmers; the initial algorithm flow was tested and iteratively remodified until a suitable flow was obtained. The adopted model was the basis for the digitalized version of the ASIC operational matrix.

Writing Codes and Creating a Suitable Platform

First, a suitable algorithm was designed, then, clear specifications for cloud and web-based support platforms were defined. Key considerations included operational effectiveness, functionality, user interface accessibility, security protocols for both users and applications, data protection measures, interactivity, and access management. Following a proper understanding of the major considerations, the codes for the digital framework were written. The process was iterative as the developer team met iteratively with the ASIC inventor to test the product at every stage. A workflow for a test-analyze-validate-and-progress model was adopted.

Producing a Proof of Concept

Following the collection of coding and testing, ASIC was made available to the developer and the expert teams who consistently interacted with the platform for over three months. During this period of interaction, bugs were identified and fixed, and web esthetics and interfacility were considered and addressed as well. Several modifications were made to ensure an optimally technically efficient ASIC product with quality attributes for access, security, reliability, interactivity, and user-friendliness in terms of attractive interface esthetics.

Quality Assurance and Validation

For the purpose of quality assurance: The ASIC inventor ran multiple tests to test operations and output and to check the product’s efficiency. An expert and senior educator was consulted for assessment, and also ASIC was made available to the public for use. Useful feedback was collected and applied.

Reflective Practice as Tool for Continuous Refining of Idea

Right from the initial release of the ASIC digital product, there has been regular analysis of feedback; much more importantly, a reflective practice based on experience, reflection, and purposeful action (ERA) has been continually applied to ASIC to optimize the technical capacities of ASIC but also very importantly, the ease of use and applications to enhance user’s productivity and efficiency in relevant contexts. This is an ever-continuous process. 

Product Improvement Through Educational Research

There is a solid plan in place for product improvement through educational research. ASIC is available for educational research purposes and concession is made to researchers to afford them the opportunity to conduct research on product sustainability and impacts. Also, there is a standard ASIC Research Instrument that can be used by researchers to conduct research in their immediate educational context which can be published eventually. On the other hand, researchers can use the instrument to collect data as members of a global ASIC EdTech consortium toward advancing medical and higher education. This questionnaire instrument is made available at no cost to researchers. 

Legal and Technical Conditions: Continuous Evaluation of Product

The ASIC (EdTech) operational matrix is published and the inventor and author have copyright to its original forms in line with the guidelines and policies of the journals in which it was originally published. The digitalized version, being a digital product, is currently being processed for a patent right. This ensures intellectual protection and protects from adulteration that could be scientifically and technically counter-productive to the optimization of the product. It also serves as a good example for educators who are equally innovators on a need to optimize their original innovations or inventions and put in place appropriate legal and intellectual protections. 

Result

The Digital ASIC Framework

**Figure 3:**
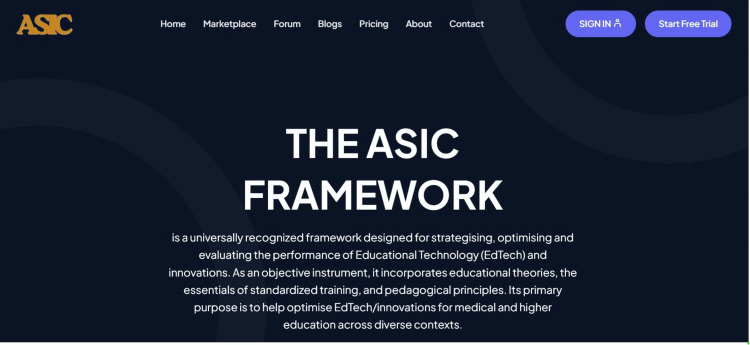
ASIC webpage interface, https://asicedtech.com/. Clicking the named buttons and icons takes the user to each feature of the ASIC EdTech framework features. The sign-in link takes the user to the dashboard that has the ASIC EdTech app with a set of instructions to follow. ASIC: Adaptation, Standardization, Integration, and Compliance; EdTech: educational technologies.

The digital ASIC framework has all the elements of the originally developed and published ASIC framework, which emphasizes Adaptation, Standardization, Integration, and Compliance in three key areas, namely, curriculum, pedagogy, and assessment. While the ASIC tenets emphasize the key areas of consideration to address when deploying EdTech and innovations, the CPA emphasizes the core domains that require attention. The digital framework and its operational matrix allow the ASIC tenets to address the CPA domains in line with each tenet. For example, the operational matrix addresses the place of adaptation in the place of curriculum, pedagogy as well as assessment. The digital framework is now available as an App that requires the user to answer 12 questions in all with three CPA (curriculum, pedagogy, and assessment) questions under each ASIC tenet (Figure 3) [35]. 

Digital ASIC Framework: A Description

**Figure 4:**
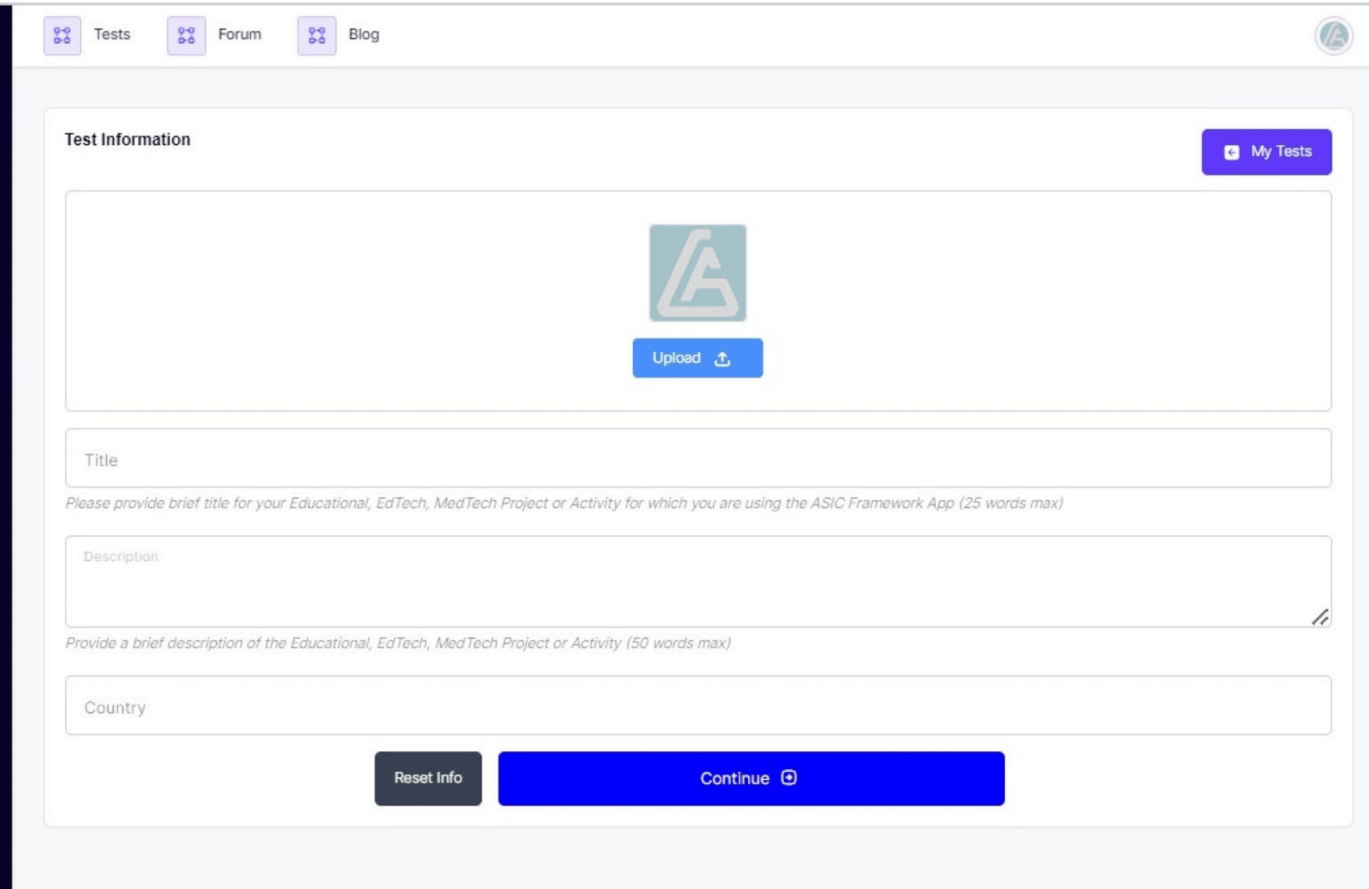
ASIC EdTech test interface. This interface allows the user to enter basic information about the technology or information of interest. This information is saved and used to identify the results of the test subsequently. ASIC: Adaptation, Standardization, Integration, and Compliance; EdTech: educational technologies.

**Figure 5:**
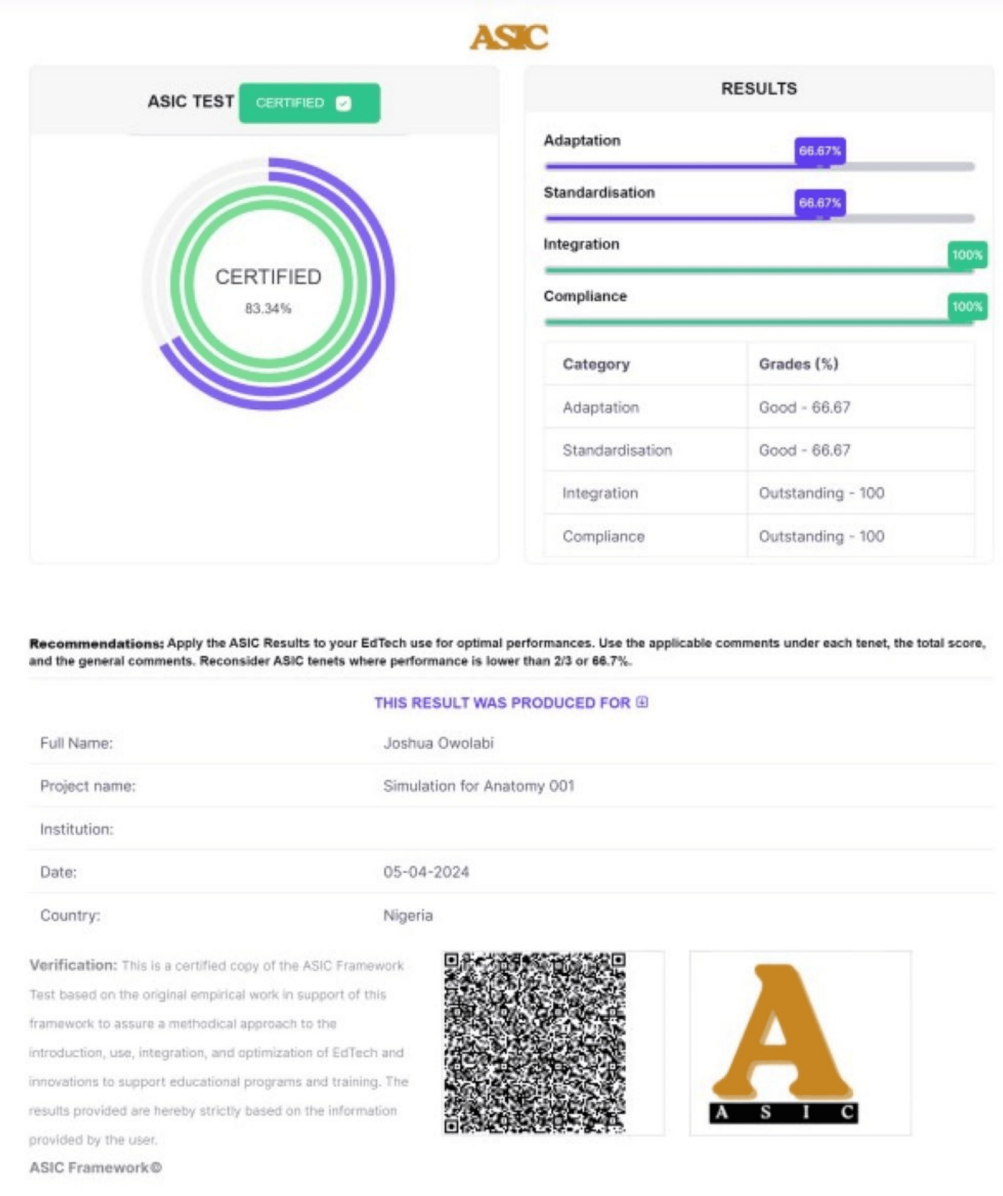
Digital results features. The result's interface has a dynamic interface with illustrations with a pictogram of test-indicated performances and a barcode.

The digital ASIC framework is accessed through the ASIC EdTech webpage or as an Android or IOS App (Figure 3). Once accessed or installed in the latter instance, the ASIC Digital interface requires the user to enter specific information about the EdTech of interest (Figure 4). A logo can also be provided. The interface thereafter requires the user to answer Yes/No to three CPA (curriculum, pedagogy, and assessment) questions under each ASIC (Adaptation, Standardization, Integration, and Compliance) tenet. Upon completion and submission, the result is generated with unique features for identity including the ASIC scores and the performance indicators both in numerical values as percentages and in graphical format. The total sum of impact value is also provided. The result is released with a barcode feature that can always be used to access the results and verify their authenticity (Figure 5). The result is also archived under the user's account and it is perpetually accessible. When downloading the unique result, the ASIC interpretation rubric is also downloadable for reference.

Explaining ASIC Working Principles

ASIC framework has four tenets namely Adaptation, Standardization, Integration, and Compliance. In terms of working principles, the ASIC operational matrix considers the application of the four tenets to three core areas of an educational experience, namely curriculum, pedagogy, and assessment. This is of key significance as the inability to operationalize the ASIC framework would either create ambiguity and subjectivity or make it impractical for accurate application. Application of ASIC to curriculum helps to address the validity and reliability of the use of EdTech and innovation for an educational experience. It also helps to determine whether the EdTech and innovation as used could provide an appropriate and adequate educational experience based on curricular requirements and whether a plan for consistent and accurate repeatability has been considered. Pedagogy also helps with the validity of an educational experience by assuring educational methods in line with relevant theories and principles such as adult learning theories. Assessment helps to measure the impact value of an EdTech/innovation on the learning experience as a measure of performance. The place of the CPA areas of ASIC applications can be highlighted as follows (see Table 2).

**Table 2:** ASIC applications to CPA. CPA: curriculum, pedagogy, and assessment; ASIC: Adaptation, Standardization, Integration, and Compliance; EdTech: educational technologies.

	CPA domains	ASIC applications
a	Curriculum	This considers the appropriateness of an EdTech/innovation to accomplish learning outcomes and ultimately program competencies-based program design and specific indicative content in the curriculum.
b	Pedagogy	This considers the educational value of the EdTech/innovation based on the applicable learning theories and pedagogical principles. It considers whether an EdTech/innovation can adequately help to achieve the objectives or outcomes of learning sessions in one or more of the cognitive, psychomotor, and affected domains.
c	Assessment	This considers whether the assessment exercises based on the use of an EdTech or innovation could meet assessment validity and reliability criteria in alignment with competencies that are required to be acquired ultimately.

Practical Use of ASIC Framework for Individuals, Groups, and Administrators

ASIC framework tool and its operational matrix can be used by individual educators, groups of educators such as those in a department or program faculty, or by administrators and organizational or institutional levels. Below are descriptions of specific contexts or settings and the basic steps included in using ASIC to assure innovation/EdTech optimization:

ASIC for individual use: When used by an individual educator, ASIC could help to make the choice of the most appropriate innovation or EdTech to acquire, how to systematically integrate the EdTech/innovation into a course or program, and how to optimize the use of the EdTech, then validly and reliably measure the impact through assessments of students and evaluation of the experience. Table 3 has the key considerations.

**Table 3:** Key steps and considerations on how to use the ASIC framework by individuals. ASIC: Adaptation, Standardization, Integration, and Compliance; EdTech: educational technologies.

	Key considerations	Additional information
1.	Identify the innovation or EdTech of interest	a. This might be made through a needs assessment
		b. It could also be a response to an identified problem that technology could address
		c. Or a response to an institutional agenda to use technology or a trend in the educational and professional arena
2.	Define clearly the context of the use	a. Determine how specifically the EdTech adds value to students’ educational experience in the knowledge, skill, and attitude domain
		b. Define the pedagogical framework that would guide the use of Edtech/innovation.
		c. Determine a practical approach to its integration into the structural and functional aspect of your educational practice or teaching by considering curricular schedules, physical or technical space, and virtual environments - whichever might be applicable.
3.	Envision its prospects to add value to your overall educational ecosystem	a. Align the use of innovation/EdTech with curriculum requirements and reflect how its use to achieve these could be justified.
4.	Apply ASIC and use the result for optimization	a. Answer each ASIC question.
		b. Use the prompt in each case to guide proper reflection.
		c. Document your supporting statements following reflections on each question.
		d. Use the document with all supporting statements as the guiding template to assure adaptation, standardization, integration, and compliance.
5.	Deploy, and measure performance by outcome, acceptability by feedback.	a. Keep to the set standard following the ASIC exercise in the previous step.
		b. Document experience in relation to the standard under each tenet.
		c. Use the experience versus pre-set standard to a reflective practice, to generate an action plan for sustainability and improvements.
6.	Improve the approach to deployment and re-deploy	a. Evaluate EdTech performance following deployment, and apply valid evaluation results.
7.	Keep optimizing	a. Apply evidence to advance practices and keep iterating.

ASIC for group use: A group of faculty members such as a member of a department or faculty members in a program can collaboratively use ASIC to guide EdTech deployment, and measure impacts and plan further improvement. In this instance, each faculty member or stakeholder can complete the ASIC Instrument, then averages of responses are computed and the interpretations of the verdicts are based on the ASIC rubrics. The alternative approach would be that the group of educators deliberate and arrive at a consensus on each ASIC Instrument item. Then, the consensus is interpreted using the rubric and this final outcome being a reflection of the collective verdict would guide decisions on EdTech use and optimization. The benefit of the latter approach is that the ASIC Instrument can be used iteratively. When an ASIC tenet scores zero, deliberations can help to reflect on the existing circumstances, and a decision can be made to restructure the same to favor a positive consideration such that the process helps to also determine the change that is required to favor the use of the EdTech/innovation of interest (see Table 4). 

**Table 4:** Key steps and considerations on how to use the ASIC framework by a team or group. ASIC: Adaptation, Standardization, Integration, and Compliance; EdTech: educational technologies.

	Key considerations	Additional information
1	Define an area of educational experience to be enhanced using innovations/EdTech	a. Define the context of use properly.
2	Identify an EdTech/innovation to adopt	a. Determine the most appropriate EdTech or innovation, especially based on need, feasibility, and educational value.
3	Design the process of introduction into the ecosystem; consider the key 4Ps namely purpose, people, process, and product/outcome.	a. Purpose - the need to use the technology should be clearly justified.
		b. People - the individuals that would be involved in the EdTech/innovation-enabled change and their roles should be clearly defined and assigned.
		c. Process - the process of change, as a roadmap, should be presented.
		d. Product/outcome - the envisioned outcome should be stated and well aligned with curricular indicative contents, program design, and professional competence to be acquired.
4	Apply ASIC tenets; obtain an initial result and use it to optimize deployment.	a. Answer ASIC questions, preferably as a group.
		b. Apply prompts to clarify verdicts leading to the answer.
		c. Generate a document of 1-3 key considerations on which a favorable answer to each tenet was premised.
		d. Carefully curate the document (generated under 4c) into a document that informs the practice that would guide the institutional and professional use of EdTech and innovation.
5	Deploy, and measure performance by outcome, acceptability by feedback.	a. Use valid and reliable outcomes to measure impact; use feedback tools to measure acceptability.
6	Analyze performance and feedback data for evidence on methods and results; re-deploy	a. Obtain evidence by properly analyzing outcomes and feedback; derive inference.
7	Keep optimizing	a. Keep improving on the methods of use.

ASIC for institutional and professional use: In the professional circle, such as the community of practice, ASIC can help to come up with standard practices or recommendations regarding the use of an innovation or EdTech especially when such is new or when an existing EdTech or innovation is being adapted or repurposed. ASIC could help in determining the educational value of such EdTech/innovation and their potential impact. Since the exercise in this instance would normally include a group of experts, the reflective processes that lead to answering ASIC questions and coming up with positive answers would equally lead to generating statements of guidelines on standard practice (see Table 5).

**Table 5:** Key steps and considerations on how to use the ASIC framework by institutions and professional bodies. ASIC: Adaptation, Standardization, Integration, and Compliance; EdTech: educational technologies.

	Key considerations	Additional information
1	Define the role of EdTech and innovations for educational purposes	a. It is important to clearly define what problem could be solved with an innovation or EdTech or what additional educational value the EdTech could contribute.
2	Characterize resources that align with educational cultures and drive change; identify EdTech and innovations	a. Based on needs assessment, context, and practical applications, identify the most appropriate EdTech or innovation of choice.
3	Apply design thinking to determine the key areas of EdTech use; establish KPIs	a. It is good to adopt a model for the design-thinking process - the IDEO model highlights steps that include Empathizing, Ideating, Concepting, Prototyping, and Implementing. What this does is to establish a roadmap for the innovative change process.
		b. Key performance indicator (KPI) helps to define the specific functions that EdTech and innovation should perform in any or all the cognitive (knowledge), psychomotor (skills), and affective (attitude) domains to make it work its use and justify resource investment that would be required.
4	Apply ASIC tool with emphasis on tenets; consider the role of the 4Ps (purpose, people, process, product/outcome)	a. Answer ASIC questions, preferably as a group.
		b. Apply prompts to clarify verdicts leading to the answer.
		c. Generate a document of 1-3 key considerations on which a favorable answer to each tenet was premised.
		d. Carefully curate the document (generated under 4c) into a document that informs the practice that would guide the institutional and professional use of EdTech and innovation.
5	Initiate change process, define milestones, measure impacts, and obtain feedback	a. Apply a suitable implementation model or strategy for the change process.
6	Analyze data and feedback; align inferences with the KPIs	a. Data on EdTech performances should be properly analyzed for evidence of impact and justification for continuous use.
7	Keep optimizing	a. Protocols should be updated continuously, and methods should be improved upon.

ASIC and the Educational Ecosystem

An educational ecosystem may be described as a system of structural and functional domains or systems interconnected by an operational network and governed by established principles and rules toward achieving an educational outcome. In a typical educational ecosystem, the structural components include physical infrastructures and the hardware that help facilitate educational experiences. This might include the physical learning spaces such as classes, laboratories, and simulation rooms; others might include machines and hardware such as computer devices, media, and specific-purpose machines such as the MRI machine in the hospital, microscopes in the laboratory, and high-fidelity mannequins in the simulation laboratories. The functional components of the ecosystem include activities that enable the use of these infrastructures and resources for facilitating sessions in classrooms, clinics, laboratories, workshops, or open fields, and other contexts of training or practice. With technologies and innovations, the networks that interconnect the structural and functional aspects of the ecosystems could be facilitated by the internet connection, or actual structural and electrical connections, aided or operated by humans or other machines. In the educational ecosystem, humans are their ultimate operators and activities are guided and aided by theories, rules, principles, and standard practices often provided as policies and guidelines.

To operationalize ASIC in an educational ecosystem, the culture of the system is of key importance. Innovations and technologies could significantly shape the culture of an educational system. It is important to carefully assess the current prevalent culture, and define the desired change to the current culture as well as the process for leading the desired change with innovations and technologies playing key roles. This is why the 4Ps become of key consideration. While ASIC could help individual members of the educational system to develop and optimize technologies and innovation, the entire organization could also use ASIC for leading change with innovations in accordance with the steps indicated for ASIC For Institutional and Professional Use as indicated in the previous section.

The basics of the ASIC algorithm include the features listed in Table 6.

**Table 6:** Basics of the ASIC algorithm and their features. ASIC: Adaptation, Standardization, Integration, and Compliance; EdTech: educational technologies.

	Basics of the ASIC algorithm	Features
1	Interface	The interface has a measuring scale that shows scores of an EdTech ASCI value; there is also a small window that shows the actual score as a percentage. There is a parallel bar that equally presents the score on a scale bar with colors showing the zone of performance with red being on the far left and the worst performance, and green being on the far right and the highest.
2	Measurement parameters	Each ASIC tenet has three considerations that are to be answered Yes/No. Each Yes response is a score of 1 out of the possible total of 12 or an equivalent percentage. See the questions on the next page.
3	Operational principles	To answer each question on the software/app, the user will have to click on the ASIC question and click the applicable option out of the Yes/No (no neutral response is allowed). Each Yes response accounts for 1 and a No gives a score of 0.
4	Prompts	The prompts include strategic questions that are aimed at stimulating ASIC users toward understanding the significance of the questions, quality reflections, and providing the most appropriate responses.

Features of the software and the operational interface are presented in Table 7. 

**Table 7:** Main parts of the software and the operational interface and its features. ASIC: Adaptation, Standardization, Integration, and Compliance; EdTech: educational technologies.

	Main parts of the software and the operational interface	Features
1	ASIC web page - landing page	The landing page has concise information that introduces ASIC as an idea and provides the meaning of its four tenets including A - Adaptation, S - Standardization, I - Integration, and C - Compliance. The Landing page has a link to the ASIC dashboard for registrations at first and subsequently log-in. The Landing page further has other very helpful information such as the frequently asked questions and links to various ASIC webpage parts such as the forum. It also has a set of frequently asked questions.
2	Dashboard	The dashboard has the link to the ASIC test software. It also has other product information and supporting links including forum, subscription, and security.
3	Access to the software	Access to the functional ASIC software is through the test link on the dashboards.
4	Operational interface	This interface registers a test; it has provisions for a logo (optional) and the test title.
5	Software operation	The software is operated by answering the questions with either Yes or No. Prompts are available for further liberation and guidance.
6	Digital result features	Upon submission, the result is downloaded automatically. A link to keep a permanent copy of the result is also automatically created. Features of a basic result include ASIC scores for a test in all sections and the overall average score and the ASIC verdict. The subscription-level results interface has a dynamic interface with illustrations with a pictogram of test-indicated performances and a barcode.
7	Interpretation rubrics	The rubric with a comprehensive guide for interpreting the result is also automatically downloaded as a PDF (see Table 8).
8	Other considerations	The dashboard has links to offerings such as ASIC courses.

Features of the ASIC framework results interpretation rubric are presented in Table 8 while the interpretations (Adaptation, Standardization, Integration, and Compliance) are provided in Table 9.

**Table 8:** ASIC framework results interpretation rubric. ASIC: Adaptation, Standardization, Integration, and Compliance; EdTech: educational technologies.

	Poor <60	Good 61-70	Very good 71-80	Excellent 81-90	Outstanding 91-100	General comment
Adaptation	Does not sufficiently satisfy at least one adaptation-related component of combined CPA requirements	Partially satisfies at least one adaptation-related component of combined CPA requirements	Sufficiently satisfies at least one adaptation-related component of combined CPA requirements	Sufficiently satisfies at least two adaptation-related components of combined CPA requirements	Sufficiently satisfies three or more adaptation-related components of combined CPA requirements	A less than 2/3 score under Adaptation requires consideration to sufficiently meet the >2/3 requirement
Standardization	Does not sufficiently satisfy at least one standardization-related component of combined CPA requirements	Partially satisfies at least one standardization-related component of combined CPA requirements	Sufficiently satisfies at least one standardization-related component of combined CPA requirements	Sufficiently satisfies at least two standardization-related components of combined CPA requirements	Sufficiently satisfies three or more standardization-related components of combined CPA requirements	A less than 2/3 score under standardization requires consideration to sufficiently meet the >2/3 requirement
Integration	Does not sufficiently satisfy at least one integration-related component of combined CPA requirements	Partially satisfies at least one integration-related component of combined CPA requirements	Sufficiently satisfies at least one integration-related component of combined CPA requirements	Sufficiently satisfies at least two integration-related components of combined CPA requirements	Sufficiently satisfies three or more integration-related components of combined CPA requirements	A less than 2/3 score under integration requires consideration to sufficiently meet the >2/3 requirement
Compliance	Does not sufficiently satisfy at least one compliance-related component of combined CPA requirements	Partially satisfies at least one compliance-related component of combined CPA requirements	Sufficiently satisfies at least one compliance-related component of combined CPA requirements	Sufficiently satisfies at least two compliance-related components of combined CPA requirements	Sufficiently satisfies three or more compliance-related components of combined CPA requirements	A less than 2/3 score under compliance requires consideration to sufficiently meet the >2/3 requirement
Total	Consider all ASIC tenets to meet minimum requirements	Consider all ASIC tenets to improve on EdTech/innovations’ performance requirements	Consider ASIC tenets with low ASIC values to improve on EdTech/innovations’ performance requirements	Consolidate ASIC tenet values by improving performance in concerned categories to fully optimize EdTech/innovations’ performance	Sustain ASIC tenets values and translate value to the actual context of use and practice for optimal EdTech/innovations’ performance	ASIC

**Table 9:** Interpretation of ASIC tenets. ASIC: Adaptation, Standardization, Integration, and Compliance; EdTech: educational technologies.

A	S
Adaptation	Standardization
Adaptation implies that innovations and educational technologies or EdTech should be suitably adapted to the learning ecosystem, program design, and institutional system, for optimal performance and best outcomes.	Standardization involves determining clearly the purpose that innovations and technologies serve, the objectives they meet; and supporting their uses with evidence for best and standard practices. It also involves the use of innovations and EdTech in alignment with sound educational and learning principles.
I	C
Integration	Compliance
Integration involves creating a place for the use of educational innovations and technology within the immediate teaching or training ecosystem, and aligning its use with other components of the educational system for optimal performance. Key considerations include system thinking and synergy.	Compliance emphasizes alignment with institutional policies, regulations, and practices as well as relevant regulatory requirements (if applicable). Evidence of compliance with institutional standards, program requirements, and regulations of relevant bodies should be addressed.
Total score = ASIC value	

## Discussion

Significance of the digitized ASIC Framework CPA operational matrix

The rapid nature of educational changes as driven by technology and innovations, coupled with a lack of requisite knowledge of educational principles that apply to EdTech and innovations coupled with a lack of practical skills to apply them has created significant heterogeneities in the methods, manners, and strategies for leading change with innovations as well as skillful and effective deployment of EdTech and innovations for optimal educational experiences. Heterogeneity, therefore, has emerged as a major problem with Edtech for educational activities. There are clear cases of heterogeneities in the types of EdTech that are available for similar educational purposes, i.e. validating the vast number of options to choose from when seeking to use tech and innovations for educational purposes. Interestingly, the eventual choices are not often premised on empirical evidence or educational values or based on guiding principles but on sentiments that bother expert opinions, availability of funds, and institutional agenda. Where existing theories and principles can be applied to guide EdTech use, many experts lack the requisite knowledge of such fundamentals or the skills and capacity to apply them in their judgments of choice and methods of use. Another source of heterogeneities is the indiscriminate use of technologies to achieve pedagogical activities that are somewhat traditional or well established without recourse to the use of evidence and application of principles to ensure the validity and reliably of the innovations or EdTech to achieve similar or better outcomes with the EdTech or innovations relative to the established principles. Often, short-term gains and immediate but arguably unsustainable results are considered as the main sources of motivation.

The ASIC framework was initially developed and has four key tenets, namely Adaptation, Standardization, Integration, and Compliance. These key tenets were organized into a functional framework, from which an algorithm was developed to optimize it for determining the validity and reliability of an educational innovation or technology to optimally provide an educational experience with emphasis on the curriculum, pedagogy, and assessment. Furthermore, the ASIC framework was matrices that could help in guiding an educator to creatively and quantitatively determine the potential impact of an EdTech or innovation within an educational ecosystem. This algorithm has been effectively digitalized. It is now available as a web-hosted software with alternative versions in the form of apps on IOS and Windows platforms. This article presents the experience as a whole. It could provide quality guidance to other educational innovators. It also serves the purpose of guiding educators on how best to optimize their innovations and EdTech for optimal learning experiences for their learners and trainees. The fact that several types of technologies and innovations can be suitably adapted and integrated into a medical or health education program using the ASIC framework is important. ASIC can guide strategic advancements with technologies and innovations in medical education with diverse products including Artificial Intelligence (AI), noting that the value of AI in medical education and care is now being seriously explored [36,37].

The problem of heterogeneities in relation to EdTech and innovations for medical and ASIC-derived solutions

There is no doubt that with the increase in advancements in technology and innovation, there will be several new types of innovations or technologies that can be used for medical education and, by extension, higher education in general. The implication of this is that there will be more varieties of tech innovations from which educators are required to choose. With more choices available, the reality of using a wide range of innovative approaches and technologies for similar purposes in different contexts of medical education will also come to light. An abundance of choice in terms of the available innovations or tech might not be a problem in itself, but heterogeneity would definitely result if standards are not set with clear guiding principles. Understandably, not all educators have extensive expertise in the primary domains of medical education in addition to their scientific and clinical competencies. The implication of this reality is that the requisite knowledge and competence to make informed decisions about the most appropriate technology and innovations in relation to the pedagogical framework to use would vary from place to place.

This is therefore why a case is being made for the use of a tested and standardized framework such as the ASIC. Not only is this framework premised on clearly stated educational theories and pedagogical principles, but it also has the versatility that supports its deployment in almost any context of medical education. It is also not cumbersome for educators to understand, especially in terms of its operational principles and applications. Clearly, digitizing this framework is also a way to democratize it and make it readily available to people in almost any part of the world. This is arguably one of the most educational frameworks with clearly listed pedagogical principles of the 21st century that address the use of innovation and technologies for medical education.

It is important to state that, unlike several other existing pedagogical frameworks that use descriptive attributes and guiding principles, the ASIC also has a quantitative approach to its use, a measure of impact, and an interpretation of the same. It is clearly a modern pedagogical framework that ranks favorably in terms of application with other popularly used pedagogical frameworks such as Kolb’s learning cycle, Argyris and Schon’s loops, Bloom’s taxonomy, and Miller’s pyramid. Nevertheless, credit should be given to the proponents of all relevant existing theories of learning and pedagogical frameworks, since they form the basis of scientific evidence to support the application and validity of the ASIC framework. It is highly recommended for individual educators, irrespective of their level of proficiency and experience, as it can objectively and consistently guide the decision-making processes regarding which type of educational technology and innovations to adopt, and more importantly, how to use such innovations or tech consistently and in line with learning theories and pedagogical principles. It is equally highly recommended to institutions and communities of practice. ASIC is arguably the only tool available currently to institutions and communities of practice to collaboratively make decisions on the best and most appropriate type of innovations and EdTech to adopt, and to justify the implementation plan by considering the four key tenets of adaptation, standardization, integration, and compliance. Furthermore, it emphasizes all the primary domains of learning, which include cognitive, psychomotor, and affective.

Also, it is important to further highlight that the problem of heterogeneity is not just about the diverse types of educational innovations and technologies that are available, but also the variations that exist in how they are used for similar purposes in different places and at different times. This aspect of heterogeneity regarding the use of EdTech and innovations can impact the qualities of validity and reliability, which are key attributes that define the assessment of learning and consequently provide justification for the acquisition of competencies. When an assessment cannot be adjudged to be valid and reliable following the use of technology and innovations, not just at the level of individual sessions or institutions but across professional settings, there arises a very important need to ensure that technologies and innovations are used in alignment with curriculum requirements, in line with sound learning theories and pedagogical principles, and validly and reliably in line with assessment principles.

ASIC remains a foremost framework that has been able to connect curriculum, pedagogy, and assessment, such that when similar technologies and innovations are used in different institutions or professional contexts, adherence to each tenet and attainment of positive indicators regarding the prospect of the innovation or EdTech in relevant domains of education and training would provide a justification for the use of technology for pedagogical purposes and justify ensuing results in assessments that have been valid and reliable. Therefore, in addition to guiding pedagogical approaches to using innovation and EdTech, ASIC also provides the basis for establishing the reliability and validity of assessments. Very importantly, this is done with a big picture of the entire program and its desired outcomes often defined by stated competencies.

Promoting a culture of best practice

One of the realities that has emerged as a result of gaps in the resources available to different institutions in various places is the significant potential gap in what constitutes standard practices across institutions that claim to train medical and health professionals. These professionals are expected to acquire similar levels of competencies at the end of their training. If the inequity that the disproportionate availability of resources to different institutions creates takes medical education back to a scenario similar to what existed prior to Flexner’s reform of the early 1900s, the quality of medical education could vary significantly across institutions because there were no regulations that set the standard for best practices [38,39].

It is important to state that products of EdTech and innovation are required to provide technical guides and manuals, in addition to commercials that promote products often supported with the best available evidence to support the usefulness of their product with a bias for positive outcomes. To their credit, some of these producers also work with technology enthusiasts as well as a selected group of tech-competent medical educators. Nevertheless, it is also important to state that these relationships do not necessarily contribute to best practices at the institutional level. Instead, they help the producers optimize their products for the best user experiences and, subsequently, patronage. Therefore, it remains the responsibility of stakeholders in medical education to set standards regarding best practices that will guide the use of innovation and tech for medical education. This aligns with calls to address both educators’ tech and pedagogical competencies in order to properly use technologies and innovation for medical education [40].

Therefore, tech-competent medical educators have a responsibility to develop innovations and present innovative ideas that are products of not just their education but also their experience in practice. They blend, weave, distill, and curate evidence available in synthesized literature, then premise it on appropriate learning theories, pedagogical frameworks, and professional standards. This process, as described, is exactly what has yielded their significant work. It is therefore important to say that the ASIC framework, as an instrument, has significant value in contributing to promoting the culture of best practices in medical education and related communities of practice. It is also important to state that other existing pedagogical frameworks that have been listed or mentioned in this article were created at a certain point in time by competent educators, and they were continually applied and oftentimes refined to support educational activities. It would appear that the development of the ASIC framework also has a place in the timeline of the history of education as a tool that could address the current trend characterized by significant adoption and use of innovations and technologies for medical and higher education.

Using ASIC to counter neo-Luddism

The Luddite movement was responsible for a major campaign against the introduction of technology into the textile industry in Europe. From this event, attempts to resist the introduction of technology into a particular domain in the years that followed this historical event have been dubbed “Neo-Luddism”. The problem of neo-Luddism has often been touted as opposition to the use of technology for medical education in certain instances, and the actors involved are often dubbed neo-Luddites or anti-technology people [41-44]. However, it is important to highlight that oftentimes people who resist technology or who are rather indifferent but unimpressed at the same time are often cautious about the negatively disruptive tendencies of certain technologies or poorly integrated innovative approaches. Even in the context of E-Health, three groups of non-adopters of E-Health technology have been identified as postponers, opponents, and critics, with the postponers and opponents groups including patients and families of patients [45]. Instances of failed attempts to deploy technology for educational purposes have reinforced resistant behavior in certain instances.

It is therefore important to understand that the use of evidence and a pedagogical framework to promote the use of technology, with an effort to not just promote standard practices but to continuously evaluate the performance of innovations and technology, could help address Neo-Luddism and provide convincing evidence to Neo-Luddites tech-ethicists. Tech ethicists are a group of people who insist that technologies are not used to break existing rules or disrupt existing orders in education. Noting that the introduction and integration of major technologies or innovations in an aspect of education could have a ripple effect, the use of a pedagogical framework can help to properly analyze the prospect and potential effects and to ensure that they are positive in terms of the impact that they might make in other aspects or domains of medical education. This argument, as presented here, is another strong indication of why the ASIC framework could be very significant to the advancement of modern medical education and should be given serious consideration by medical educators and other stakeholders, including academic leaders. People’s appreciation for the use of technologies in medicine currently varies significantly; with better education, exposure, and promotion of best practices, educators, professionals, and patients will increasingly appreciate the value of technologies [46].

Importance of ASIC to stakeholders - educators, administrators, instructional designers, and academic leaders

Much of the case that has been made for a need to standardize the use of EdTech and innovation has emphasized the importance of a standard framework for the job of medical educators. Administrators can also be better guided in making decisions on the choice of technologies to procure and in developing a plan to integrate technologies and innovation into the educational ecosystem while assuring value for the investment of resources and capital. For instructional designers, it is important for them to consider the specific innovation or technology that is being introduced and the impact of the same on the existing educational and infrastructure ecosystem. For example, it might be important to consider how a new technology, such as educational software, might be integrated into the existing learning management system with optimized access provided to the student. It might also be important to consider how other resources that are already in existence will be functionally connected with a newly introduced technology or innovation such that they can run in sync for an optimal student experience. More than ever before, instructional designers have to consider not just the prospect of a particular edtech or innovation based on its performance, but also its synchronization with other components of the educational ecosystem.

Academic leaders are often required to ultimately make decisions regarding the innovations and technologies to be procured for their various programs or groups of students. There are times when they are required to consider cases or even arguments as presented by educators and other stakeholders. Using a framework such as the ASIC can guide all these stakeholders in working collaboratively to arrive at the best possible decisions by aligning paradigms, while at the same time considering the value of technologies and innovations based on their potential educational value or impact. For example, it is possible that a newly developed sophisticated technology might not be adding significant value to the program based on competencies that are required of students, even when the proponents are quite enthusiastic about its futuristic value. A practical framework can therefore help an academic leader to judge innovation and technology based on its value for money and return on investment, in addition to the actual educational value. Should there be an instance where an institution chooses to be a trailblazer by pushing the boundary of training by considering the use of a particular technology or innovation that is not already aligned with stated competencies to be acquired through a program, a standard framework such as the ASIC might still be able to help in carefully measuring what is to be committed to such effort while ensuring that it is not done at the expense of the already established and identified outcomes, which might be used by regulators and institutional standards to judge the success of the program? Academic leaders, especially, should also be aware that a major way to capitalize on the value that Edtech and innovation could add to the future of medical education is to start enshrining a healthy culture of technology into medical education right from now. Future doctors and health workers would practice in a tech-enriched environment where they need to be exposed adequately to technology during their training [19,47,48].

## Conclusions

EdTech and innovations including educational devices and AI have significant roles in delivering medical education. The vast variety of EdTech and innovations available to educators as well as the different pedagogical approaches to using the EdTech and innovations have created a problem of heterogeneities that could undermine standardized educational experiences. There are currently no major tools and frameworks to guide EdTech deployment and optimization, hence the place of ASIC as a foremost framework for guiding the deployment of EdTech or innovations and their optimization. The ASIC framework has four tenets including Adaptation, Standardization, Integration, and Compliance. This article has presented the process of digitalizing the ASIC framework as an innovation. It has also provided provided information to guide users.
